# Supplementary material for: Are carbon emissions trading and green financial instruments synergistic? -Comprehensive quantitative research based on content analysis
Source: PLoS One. 2024 Mar 7;19(3):e0298601. doi: 10.1371/journal.pone.0298601 (PMC10919601; doi:10.1371/journal.pone.0298601)
Supplement: S1 Data — (ZIP) [file pone.0298601.s001.zip › Data/地级市Z_F工作报告.zip!/╔┬╬≈/╤╙░▓╩╨/└┤╫╘╣1⁄2╓┌║┼í╛┬φ┐╦╩2╛▌═°í┐_2019.docx]

各位代表：

我代表市人民政府向大会报告工作，请予审议，并请市政协委员和其他列席人员提出意见。

一、市五届人大三次会议确定任务完成情况

2018年，是全面贯彻党的十九大精神的开局之年，也是延安转型发展、追赶超越进程中极不平凡的一年。全市上下坚持以习近平新时代中国特色社会主义思想为指导，在市委的坚强领导下，坚持新发展理念，落实“五新”战略任务，突出“三大攻坚战”，扎实做好“有中生新”“无中生有”两篇文章，统筹稳增长、促改革、调结构、惠民生、防风险各项工作，取得了一批引领未来的开创性成果，破解了一批制约发展的难题，办成了一批惠及广大群众的民生实事，较好地完成了市五届人大三次会议确定的目标任务。全市完成生产总值1558.9亿元，增长9.1%；地方财政收入148.3亿元，增长5.6%；全社会固定资产投资增长6.9%；城乡居民人均可支配收入分别增长8%和9.2%。经济运行总体平稳、稳中有进、进中向好，发展速度创2013年以来最高，继2007年以来首次超过全省平均水平，一举扭转连续九年全省垫底被动局面。

（一）经济保持较快增长。出台工业稳增长促投资推动高质量发展15条措施，生产原油1529万吨、加工原油924万吨，生产煤炭5112万吨、天然气50亿立方米、LNG80万吨，发电70亿千瓦时，能源产品产量全部实现正增长。“延安”牌中支卷烟投放市场，生产卷烟32.2万箱，产值增长0.8%。规上工业增加值增长10.4%，较上年提高3.4个百分点。洛川县现代农业产业园被认定为首批国家级现代农业产业园，建成市级现代农业示范园17个和市级“一村一品、一乡一业”专业村230个、专业镇18个。洛川老庙镇、吴起五谷城镇桐寨村被认定为第八批全国一村一品示范村镇。21家农民专业合作社跻身国家示范社。粮食总产69.6万吨。新建果园14万亩，苹果总产289万吨，新建智能选果线50条、冷气库12.1万吨、品牌直销店105个，“延安苹果”标准正式发布，苹果产业后整理成效显著，在遭受冻灾、产量下降的情况下，带动果农人均增收2510元。生产蔬菜108万吨，增长6.3%。畜禽饲养量和肉蛋奶产量稳定增长。非洲猪瘟有效防控，“大棚房”清理整治取得阶段性成果。成功举办延安首届中国农民丰收节。全域旅游示范市、红5A景区创建和中国革命文艺家博物院、圣地河谷二期、“锦绣黄龙”、甘泉大峡谷等项目快速推进，壶口瀑布、乾坤湾等景区一展新颜，“圣地延安数字博物馆群”吸睛点赞。接待游客6344万人次，旅游综合收入411亿元，分别增长25.4%和37.5%。红色教育培训29.3万人。社会消费品零售总额增长10.7%。

（二）结构调整多点突破。《延安综合能源基地发展规划》加快实施，富县煤油气资源综合利用、洛川轻烃裂解、大唐热电联产等一批助推转型升级的重大项目建成投产，华为云计算数据中心投入运营，高新区金风风机制造、安塞LNG等项目投入生产，彩虹光伏玻璃、子长500万吨低阶煤多联产循环综合利用中试装置具备生产条件，延长石油伴生气资源综合利用项目快速推进，华能延安电厂全面复工，陕北至湖北电力外送通道获批，陕北至关中第二通道750千伏输变电工程全线贯通。中航油、三达膜科技和中大门网络科技成功落户。战略性新兴产业增加值增长12.4%。17个县域工业集中区实现营业收入489亿元，增长19.6%。延安基金小镇开业运行，898商品交易中心产品上线交易，新增延安光谷、腾讯众创空间等6家创新创业平台，新经济企业成倍增长，累计达到445家。

（三）脱贫攻坚成效显著。举全市之力、以必胜信念、下绣花功夫，推动脱贫攻坚取得历史性突破，实现贫困村驻村工作队和贫困户帮扶责任人全覆盖，贫困村集体经济、贫困户产业、兜底保障全覆盖，农村基础设施全面提升。全年投入各类扶贫资金60.9亿元。“八个一批”脱贫工程扎实推进，扶持引导贫困户融入苹果、棚栽、养殖、旅游等产业链，贫困户苹果免费上线分拣和入库贮藏，“龙头企业+合作社+村集体经济+贫困户”模式有效推广。4950户易地搬迁户分房到户，移民搬迁工作居全省第一方阵。7409户危房改造竣工率、入住率、达标率均达100%。新修农村公路446公里，维修“油返砂”道路570公里，完成571处安全饮水提升工程，建制村全部通硬化路、通动力电，农村饮水安全整体达标。实施中央定点扶贫、苏陕扶贫协作、国企合力团等社会扶贫项目1000多个。延长县成功“摘帽”，完成剩余338个贫困村退出、3.7万贫困人口脱贫，宜川、延川脱贫退出各项指标达标，贫困发生率降至0.66%，全市实现整体脱贫。荣获全国脱贫攻坚组织创新奖。

（四）城乡建设步伐加快。新区人民公园建成开放，北大培文学校当年建设当年招生，酒店餐饮、生活服务、建材家居等商圈正在形成，一批高品质住宅成为群众改善住房条件的理想选择。完成西北局旧址周边改造，建成开放延河情公园、黄土情公园，宝塔山景区保护提升工程主体完工。小砭沟至肖林环线、方塔至东过境道路建成通车，西沟桥、南滨路拓宽改造和双拥大道综合管廊建设全面完成。火车站周边环境综合整治、中心巷改造提升等8个城市“双修”项目快速推进，试点经验得到住建部肯定。高新区万达红色旅游小镇正式落地。南泥湾开发区红色教育培训及基础设施配套项目加快实施。全国文明城市、国家生态园林城市和国家节水型城市创建有序推进。延川、洛川、甘泉成功创建省级卫生县城，实现省级卫生县城全覆盖。黄龙荣获“省级森林城市”称号，洛川荣获“省级园林县城”称号。延川文安驿被列为全国特色旅游城镇，黄龙瓦子街、志丹永宁荣获“陕西省旅游特色名镇”称号。宝塔区冯庄乡康坪村荣获“中国美丽乡村”称号，累计建成省级美丽宜居示范村30个，农村人居环境综合整治工作受到国务院通报表彰。全年投入各类污染防治资金27.6亿元。狠抓中省环保督察反馈问题整改，建成投用姚店污水处理厂和28个重点镇污水处理设施、24个垃圾填埋场，主要河流劣Ⅴ类水质基本消除，城区空气质量优良天数达到315天，位居全省第三。荣获“2018中国美丽山水城市”称号。

（五）基础保障更加有力。市级重点项目开复工率达96%，创历年新高，完成年度投资计划的105%。民间投资增长96.1%，位居全省前列。延安南泥湾机场建成通航。西延高铁正式开工。吴定高速建成通车，清涧至子长等7条高速公路加快建设。北过境、新机场连接线、延延高速连接线二期、黄延二通道枣园出口连接线、志丹至南梁红色旅游公路、延长至马头关二级公路改建工程建成通车。宝塔区被命名为全国“四好农村路”示范县（区）。黄河引水工程实现向市区供水，龙安水库、王瑶水库扩容工程前期有序推进。延河滨水景观带成为市民休闲好去处。治沟造地12.5万亩。新造林81万亩，三北防护林工程成为全国生态保护修复示范样板。延安东330千伏输变电工程进展顺利。“宽带中国”建设任务提前两年完成，城乡光网实现全覆盖。

（六）改革开放纵深推进。引导退出煤矿5处，淘汰落后产能105万吨。减税降费7.2亿元。46户国有企业改制圆满收官，市属经营性国有资产实现集中统一监管，启动51户市属国有企业公司制改制，与中航油、陕建集团、英雄互娱等组建混合所有制企业，延安水务、建投集团挂牌成立。被确定为全国工程建设项目审批制度、营商环境试评价城市和全省相对集中行政许可权改革试点，市县行政审批服务局全部组建，审批时限平均压缩65%，市级政务服务事项网上可办率达80%以上，企业群众办事更省心、更舒心、更暖心。荣获首批“全国守信激励创新奖”。40家企业在陕西股权交易中心挂牌，引进江苏必康制药，实现上市公司“零”突破。新增市场主体4.3万户、“五上”企业230户，非公经济占GDP比重达到29.3%。13个县级农村产权交易中心建设实现全覆盖，1784个行政村完成清产核资，土地流转率达到29.8%。实行政府债务限额管理，严厉打击非法集资、金融诈骗等违法犯罪活动。金融机构各项存款余额1610亿元、增长2.8%，贷款余额1187亿元、增长6.9%，存贷比达73.7%，排名全省第二。城市管理执法体制改革基本完成，机关事业单位养老保险制度改革走在全省前列，企事业单位公务用车制度改革全面完成，国有林场改革通过省级验收。加快发展“三个经济”，新增至深圳、海口、天津、厦门航线航班，入选国家陆港型物流枢纽承载城市名单，与西安、广州、南昌缔结友好城市，与雄安、赣州等地达成友好合作意向，中国延安·格鲁吉亚产业园商务馆开馆在即。实施招商引资项目386个，到位资金476.7亿元，增长15.7%。

（七）民生福祉持续改善。用心解决群众在出行、教育、医疗、养老、住房等方面的操心事、烦心事，民生支出321亿元，增长11.3%。新增城镇就业1.6万人，城镇登记失业率3.5%。为农民工追讨工资9600多万元。城乡低保标准稳步提高，发放高龄补贴1.2亿元、困难群众救助补助金11.1亿元。取消城乡居民门诊统筹各病种起付线，政策性报销比例达77.4%。棚户区改造新开工3.2万套，分配公租房7729套。“民生十件实事”扎实推进，建成果蔬超市12个、早餐门店50个、公厕184座、停车楼4处，建设便利加油站8座。新建标准化示范社区7个。延安大学新校区建成招生。投资10.4亿元，新建、改扩建中小学、幼儿园39所，新增学位近2万个，普惠性幼儿园占比达到85%，有效缓解了入学难、大班额问题。延安第二医疗集团组建运行，市中医医院迁建项目建成投用并与北京大学第三医院合作办医，市精神病医院基本建成，卓雅妇幼儿童医疗综合体启动建设。获评首届“健康中国”年度标志城市。陕北说书馆主体完工，基层综合性文化服务中心达标率达到69.6%。西部地区国家级文化生态保护实验区建设现场经验交流会、首届民歌艺术节成功举办，“延安过大年”被评为第四批国家公共文化服务体系建设示范项目。成功举办国际雪联中国城市越野滑雪积分大奖赛首站赛事。芦山峁新石器时代遗址成为2018年中国六大考古新发现之一。市级续修志《延安市志（1997-2010）》率先在全省出版发行。深入开展扫黑除恶专项斗争，打掉黑社会性质组织1个、恶势力犯罪集团10个、恶势力犯罪团伙62个，刑事治安案件发案“双下降”。安全生产“三项攻坚”行动扎实推进，四项指标分别下降16.5%、4.6%、10.9%和52.1%。食品药品安全保障水平明显提升。积极推进阳光信访、责任信访和法治信访，赴省进京上访“双下降”。黄龙“普法责任田”模式得到司法部肯定，新时代“十个没有”工作经验全省推广。

（八）政府自身建设不断加强。认真贯彻落实习近平新时代中国特色社会主义思想，依法履职尽责，自觉接受人大、政协和社会各界监督，落实《延安市人民代表大会关于全力推进延安苹果产业后整理的决定》和《延安市退耕还林成果保护条例》，115件人大代表建议和303件政协委员提案全部办结。严格落实中央八项规定实施细则精神和省市实施办法，扎实开展“以案促改”，坚决纠正“四风”。国家深化扶贫领域腐败和作风问题专项治理工作推进会在我市召开。持续压缩一般性支出，部门日常办公经费压缩15%，会议费、车辆运行费均压缩10%。网民留言和“12345”市民热线办结率分别达到98.2%和99.3%，人民网网民留言办理获得全国先进。政府决策科学化、民主化、法治化水平不断提升，被省政府授予建设法治政府示范市。

统计、人防、双拥、盐务、气象、档案、慈善、助残、民族宗教、妇女儿童、外事侨务、国家安全、国防动员和后备力量建设等工作都取得了新成绩。

各位代表，一载风雨砥砺，一载春华秋实。过去一年，全市稳的基础在巩固，进的动力在增强，好的因素在积累，成绩来之不易。这是市委坚强领导的结果，是市人大常委会、市政协监督支持的结果，是全市人民团结拼搏的结果。在此，我代表市人民政府，向全市广大干部群众，向各民主党派、工商联、无党派人士和离退休老同志，向中省驻延企事业单位、驻延部队、武警官兵和公安民警，向各位人大代表、政协委员和社会各界人士表示崇高的敬意和衷心的感谢！

在总结成绩的同时，我们也清醒地看到，经济社会发展中还存在不少困难和问题，主要是：结构调整任重道远，经济下行压力较大，新动能培育效应尚未成势，非公经济发展缓慢；谋划储备的大项目、好项目少，投资增长后劲乏力；区域、城乡发展不平衡，基础保障能力有待提高；教育、养老等公共服务供给不充分，一些民生实事推进不够理想；脱贫内生动力还需增强，巩固成果长效机制有待完善；环境污染防治任务依然繁重，节能降耗形势不容乐观；部分政府工作人员思想观念、法治意识、专业素养、担当精神与新时代新要求不相适应。我们将采取有效措施，切实加以解决。

二、2019年经济社会发展预期目标和主要任务

2019年是新中国成立70周年，也是决胜全面建成小康社会的关键之年。延安发展面临难得历史机遇，也存在诸多挑战。世界经济增长低迷态势仍在延续，国内经济运行稳中有变、变中有忧，下行压力持续加大。但更应看到，我国经济长期向好的基本面没有改变，国家坚持稳中求进工作总基调，强化宏观政策逆周期调节，实行积极的财政政策和稳健的货币政策，加大减税降费力度，进一步支持西部大开发，加快基础设施补短板，激发微观主体活力；省委、省政府全面落实“五新”战略任务，大力发展“三个经济”，更加注重区域协调，推进陕北转型持续发展。中省一系列政策利好，为我们抢抓机遇、加快发展提供了舞台、开辟了空间。只要我们保持定力，增强信心，精确识变，顺势而为，就一定能够掌握发展主动权，推动全市经济社会发展迈出新的坚实步伐。

今年工作的总体思路是：以习近平新时代中国特色社会主义思想为指导，统筹推进“五位一体”总体布局和协调推进“四个全面”战略布局，紧扣追赶超越定位和“五个扎实”要求，贯彻落实省委“五新”战略任务和市委五届六次全会精神，深入实施“五大发展战略”，坚持稳中求进工作总基调，坚持新发展理念，坚持推动高质量发展，坚持以供给侧结构性改革为主线，坚持深化市场化改革、扩大高水平开放，大力发展“三个经济”，继续打好“三大攻坚战”，统筹推进稳增长、促改革、调结构、惠民生、防风险各项工作，切实增强人民群众的获得感幸福感安全感，努力让延安人民生活得更幸福更美好。

主要预期目标是：生产总值增长8%左右，全社会固定资产投资增长10%左右，地方财政收入增长8%左右，城乡居民人均可支配收入分别增长8%和9%。重点做好八个方面工作：

（一）供给需求双向发力，促进经济平稳较快增长。落实“巩固、增强、提升、畅通”八字方针，供给推动、需求拉动，稳住经济增长基本盘。

加速释放工业产能。强化与中省企业协调对接，加大能源勘探开发力度，扩大卷烟生产和代加工量，推动主要工业产品产量稳定增长。生产原油1570万吨、加工原油965万吨，生产煤炭5200万吨、天然气60亿立方米、LNG95万吨、卷烟40万箱，发电70亿千瓦时。加速释放大唐热电联产产能，力促富县煤油气资源综合利用、液化空气工业气体、洛川轻烃裂解、彩虹光伏玻璃、志丹和安塞LNG等项目规模化生产，争取延长石油伴生气、子长羊马河煤矿、车村煤矿一号井、三达膜科技等项目早日建成投产。规模以上工业增加值增长8%。

扩大投资促进消费。发挥有效投资对优化供给结构的决定性作用，抓住国家基础设施领域补短板机遇，用好延安金字招牌，主动申报争取，推动更多项目资金落地。改进招商方式，精准招商、产业项目模块招商，提高项目履约率和资金到位率。积极吸引扩大民间投资，规范政府和社会资本合作（PPP）模式，形成投资主体多元格局。落实领导包抓、清单管理、动态监管、定期会商制度，加快项目建设进度，提高项目投资强度。发挥消费对增长的基础性作用，在旅游、文化、体育、健康、研学、房产、汽车等领域培育壮大消费热点，提升消费能力，促进消费供给升级，社会消费品零售总额增长10%。

发展壮大实体经济。落实减税降费和工业用电价格下调政策，扩大直供电交易规模，降低企业生产经营成本，支持延安能化、旅游、城投、水务、建投集团做大做强，推进实体经济强动力增活力。出台支持民营经济高质量发展的意见，建立政府与金融机构、小微企业定期沟通交流及政银企对接、财政风险补偿、信贷担保等机制，落实对小微企业和科技型初创企业的普惠性税收减免政策，建立民营企业纾困基金，开展违约拖欠中小企业款项清理行动，有效缓解企业融资难融资贵问题。引导广大民营企业家聚精会神办企业、遵纪守法搞经营，依法保护民营企业家人身财产安全，推动民营企业健康发展，使民营企业成为延安转型发展的新亮点、高质量发展的增长极。新增市场主体4.5万户，培育发展“五上”企业200户，非公经济占比提高2个百分点以上。

（二）持续做好“两篇文章”，加快培育发展新动能。统筹推进产业链、创新链、资金链、政策链有机融合，“有中生新”“无中生有”，努力构建多元支撑、多极增长、链条高端的现代产业体系，提升发展质量效益。

着力推动高端能化。深入实施《延安综合能源基地发展规划》，建成黄陵120万吨煤炭洗选、延川20万吨可发性聚苯乙烯、延炼50万吨航煤油生产技术改造等项目，加快推进华能延安电厂、500万吨低阶煤多联产循环综合利用、天然气一体化全产业链开发、志丹LNG二期等项目，开工陕北至湖北电力外送通道、陕煤黄陵电厂、延长石油富县电厂、安塞热电联产、高新区智慧能源、60万千瓦新能源、吴起20万吨和宜川40万吨LNG等项目，积极推进以延安为起点的电力外送通道建设，启动与延长石油集团谋划的四大类13个重点项目前期，推动能化向产业链中高端加速跨越。

着力培育新兴产业。实施创新驱动发展战略，发挥院士工作站、重点实验室、工程技术研究中心、产业技术创新联盟等平台作用，精准对接企业发展需求，推动科技创新成果转化。加快推进延安必康现代医药智慧健康综合体、宝塔新材料高新技术产业园、安塞石墨烯电热膜生产、高新区天才科技艾眼产品、碳纤维研发生产和翰墨高端包装等项目，战略性新兴产业增加值增长12.5%。落实鼓励新经济发展政策，落地新经济企业营业收入达到50亿元以上。

着力促进文化旅游产业升级。以全域旅游示范市创建为统领，加快文化旅游“五大板块”“六大园区”建设，完成红5A景区创建，实施革命旧址群保护利用规划，完善景区景点服务功能和基础设施配套，建成宝塔山游客服务中心和黄陵、宜川、黄龙、延川县级游客服务中心，加快推进黄帝陵国家文化公园、黄河秦晋大峡谷文化旅游长廊、甘泉志丹“红谷”丹霞地质遗迹景观走廊、安塞黄土风情文化产业园、南泥湾红色文化小镇等重大项目。策划筹办有影响力的论坛、赛事、节会，推出精品旅游线路，开发特色文创产品，促进文化旅游产业内涵式增长，接待游客、旅游综合收入分别增长14%和20.5%。规范红色教育培训，科学设置课程，完善教学设施，合理调配师资力量，推动高质量规模扩张，全年培训40万人，增长36.5%。

着力加快现代服务业发展。实施现代服务业创新发展三年行动计划，加快安塞、青化砭等物流园区建设，建成快递物流等6个物流园区，深化与中国供销集团、青岛巴龙等企业合作。出台支持住宿餐饮业发展政策措施，加快推进新区地建集团酒店群、延安国际会议中心等项目建设。大力发展电子商务、融资租赁、节能环保、研发设计、区块链技术等新兴服务业态，促进生产性服务业向价值链高端延伸，生活性服务业向高品质转变。第三产业增加值增长9%。

（三）实施乡村振兴战略，推动农业农村优先发展。按照“产业兴旺、生态宜居、乡风文明、治理有效、生活富裕”总要求，实施《延安市乡村振兴战略规划》，在实现农业农村现代化征程上迈出新步伐。

大力发展现代农业。坚持绿色兴农、品牌强农，加快推进农业由增产向提质转变。粮食产量稳定在70万吨。新增果园6万亩，生产苹果320万吨。蔬菜产量达到110万吨。建设规模化养殖场60个，保持肉蛋奶产量稳定增长。支持红枣、核桃、花椒、中药材、油用牡丹、地椒羊、中蜂等特色种养业发展。启动建设乡村振兴试点示范村35个、现代农业示范园20个，加快创建出口农产品质量安全示范区、生态原产地保护产品示范区和良好农业规范认证示范区。深入推进以苹果为主的特色农业产业后整理，再建一批智能选果线和冷气库，推行农产品标准化生产和质量追溯，加大“延安苹果”“洛川苹果”宣传营销力度，提高品牌影响力。新认定市级龙头企业20家，发展农民专业合作社50家、家庭农场100个。推行苹果“银行+期货+保险”模式，健全完善农业技术服务和防灾减灾体系，做好非洲猪瘟等各类疫病防控。建成智慧农业综合信息平台。鼓励农业与旅游、电商、康养等业态融合发展，促进农业“接二连三”。

推进美丽乡村建设。落实农村人居环境整治三年行动方案，编制县域乡村振兴规划，按照“短期抓清理、中期抓治理、长期建机制”的思路，持续完善水电路气讯等基础设施和科教文卫体公共服务设施，实施“三清一改”行动，提高农村面源污染治理水平，主要河流沿线、水源保护区、形成污水径流的村庄全部建成污水处理设施，85%以上的村庄实现生活垃圾分类、收集和转运处理，规模化畜禽养殖场粪污综合利用率达到73%以上，卫生厕所普及率达到70%以上，10%的行政村建成美丽宜居示范村，打造一批农村人居环境治理集中连片示范区。

激活乡村振兴内生动力。深入推进农村集体产权制度改革，集中开展清产核资检查验收，继续抓好集体经济组织成员界定和股份制改革，巩固产权制度和“三变”改革成果。建成市县乡农村产权交易平台，村集体经济组织比例达到85%，逐步消除集体经济“空壳村”。推进农业水价综合改革试点。用足用好城乡建设用地增减挂钩节余指标省域流转政策，盘活农村存量土地，拓宽移民搬迁基础设施、产业园区建设和现代农业发展融资渠道。

加强乡村有效治理。弘扬社会主义核心价值观，做好新时代文明实践中心试点工作。健全完善自治法治德治相结合的乡村治理体系，发挥村规民约作用，坚决整治婚丧嫁娶大操大办、盲目攀比、聚众赌博、封建迷信等陈规陋习，引导群众移风易俗，培育文明乡风、良好家风、淳朴民风，营造和谐包容、安定有序、向上向善良好环境，让广大农民享有更有品质的生活。

（四）坚守阵地久久为功，打赢打好三大攻坚战。坚持既定目标不动摇，聚焦靶心，精准施策，持续发力，巩固提升成果，打好重点战役，夯实决战决胜基础。

全力巩固精准脱贫成效。坚持帮扶力度不减、支持力度不减、投入力度不减和政策的连续性稳定性，重振精神再出发，扎实推进脱贫成果巩固“十大提升行动”，扭住产业扶贫这个关键，让贫困户深度融入产业链，全面提升“造血”能力。易地扶贫搬迁安置户10月底前全部入住，旧宅基地腾退任务全面完成。加强农村公路、安全饮水设施运营管理。引导社会力量持续加大帮扶力度。建立信息监测、警示预警机制，动态掌握贫困状况，及时跟进帮扶措施，提升脱贫质量。研究解决收入水平略高于建档立卡贫困户群体缺乏政策支持问题，确保小康路上不漏一户、不落一人。

突出抓好污染防治。汲取秦岭北麓西安境内违建别墅问题教训，抓好中省环保督察反馈问题整改，全力打好青山碧水蓝天净土保卫战，让延安人民享有更多环保福利、生态红利。加快国土绿化，造林50万亩。加大封山禁牧力度，巩固退耕还林成果。严控开发建设范围，加强桥山、子午岭、黄龙山等森林保护，严禁乱挖山体、非法采石破坏植被，巩固“大棚房”清理整治成果。加强水污染防治，推进县级以上水源地规范化建设，压实各级河长管水、治水、护水责任，启动城镇垃圾污水处理提质增效三年行动，国省控断面水质全面达标。落实减煤、控车、抑尘、治源、禁燃、增绿举措，城区空气质量优良天数达到318天。强化土壤污染防治，加强危险废物规范化管理和整治。大力发展循环经济，建成餐厨垃圾资源化利用和无害化处理场。推进节能降耗，单位GDP能耗下降2.9%。

坚决守住防范化解重大风险底线。坚持堵“后门”与开“前门”并重，牢固树立风险防范意识，强化政府性债务风险管控，实行负面清单管理，建立政府举债终身问责制，加大存量债务置换力度，严格控制增量，逐步消化存量。稳步推进投融资平台公司市场化实体转型。加强重点行业、企业信贷风险排查，强化融资性担保机构、小额贷款公司监管。严厉打击非法集资、非法揽储等违法违规金融活动，严密防控民间融资、各类交易场所、互联网金融等风险，守牢不发生系统性金融风险底线。

（五）充分发挥后发优势，促进城乡区域协调发展。激发城镇化和基础设施建设空间大的后发优势，加快推进区域协调、城乡一体。

提升中心城市品质。坚决贯彻落实习近平总书记重要指示批示精神，防风险、补短板、强弱项，兴利除弊，高质量建设延安新区，构建产城融合、人与自然和谐共生的宜居宜业宜游现代生态家园。加快新型城镇化综合试点和城市“双修”、城市设计试点，推进中心城市减脂增肌、有机更新。抓好以高速公路出入口、中心街、延河为重点的“四口三街两河”城市“双修”，加快中心街地下综合体和长青路周边、延烟路山体等棚户区改造。新建火车站快速通道二期等道路，增加公交频次和停靠港湾，加强出租车、网约车管理，用2至3年时间有效解决城区交通拥堵问题。推进全国文明城市、国家生态园林城市和国家节水型城市创建，加快智慧城市建设，完善市政配套设施，强化市容市貌综合整治，提高城市治理水平。

增强城镇承载功能。依托区位优势和发展基础，完善县城发展建设规划，打造产业集聚度高、人口承载力强、功能设施完善、辐射集镇乡村的新型县城。推进子长撤县设市，启动黄陵、子长县城“双修”。支持重点镇、文化旅游名镇完善基础设施和公共服务设施，强功能、增效能、提品质，促进农业人口转移。

加快补齐基础设施短板。推进城乡基础设施互联互通，建成绥德至延川、清涧至子长高速，加快延长至黄龙、吴起至华池等5条高速公路和西延高铁建设，推进延安东西高速公路连接线前期，开工延榆高铁、新机场南连接线、南泥湾过境公路等项目。加快推进黄河引水后续工程，增强城市供水保障能力，促进水源、输水、供水一体，构建现代水务体系。推进龙安水库、王瑶水库扩容、永宁水库、白龙江引水等项目前期。加快延安东330千伏输变电、蒙华铁路供电工程建设，有序推进电网基建和城农网改造，实现重点区域“N-1”供电，县城、重点镇、园区110千伏电网全覆盖。加快县域坚强电网建设，增强新能源电力消纳外送能力。推动行业部门数据横向耦合，实现信息数据资源共享。

促进区域协调发展。大力发展县域经济，加快推进“三区九园”和17个县域工业集中区建设，新增入园企业30户，营业收入增长12%。提升县区农业示范、现代物流、文化产业等园区效益，推动县域经济争先进位。统筹推进区域板块联动，加快新区、高新区、南泥湾开发区建设，形成主业突出、错位发展格局。抓好新区新经济产业园、北航科技园和中央商务区、中大门保税店等项目建设。加快高新区万达红色旅游小镇和绿色建材循环利用产业园、创新科技产业园建设，争取升级为国家级高新技术产业开发区。建成南泥湾教育基地、大生产广场、国家湿地公园等项目，再现“陕北好江南”。加快延塞快速干道、新动能产业园建设，强化功能互补，推进安塞与延安中心城区一体发展。统筹推进沿黄公路沿线城镇建设、产业发展、人居环境整治，打造沿黄城镇带、产业带、生态景观带。

（六）系统整体协同推进改革，全方位扩大对外开放。站在更高起点深化改革开放，充分激发发展动力和创新活力，不断提升竞争优势。

聚焦重点领域改革。深入推进国资国企改革，完成51户市属企业公司制改制，推进国有资本授权经营体制改革，改组或组建国有资本投资运营公司，加快混合所有制改革，做强做优做大国有资本。全面实施预算绩效管理，深化市以下财政事权和支出责任划分改革。加快中航油、英雄互娱、三达膜科技等企业上市，支持优质企业发行债券，利用多层次资本市场，增强金融服务实体经济能力。稳步推进水价改革，制定同城同价、分质分类供水办法，逐步实现水电气暖“多表合一”。统筹推进医疗、文化、供销等领域改革。

优化提升营商环境。深化“放管服”改革，加快推进相对集中行政许可权、工程建设项目审批制度改革试点，在精简审批事项和申报材料、优化流程、压缩时限上持续用力，取消企业银行账户开户许可，加快“多规合一”“多评合一”“多测合一”“多图联审”“区域评估”实施进度，完善“互联网+政务服务”平台功能，行政审批服务事项线上“一网通办”、线下“只进一扇门、最多跑一次”，催生市场主体、增加民间投资、降低办事成本。厘清审管职责，提升服务水平，推进行政审批与事前事中事后监管无缝对接。坚持重心下移，同步推进市县改革，在“国字号”改革试点上创出新经验、打造新模式。

推动更高水平对外开放。以开放合作拓展发展空间，主动融入“一带一路”，强枢纽、扩门户、促流动，推动人流、物流、能量流、信息流、资金流等要素畅通融合，加快“三个经济”发展。抓住入选国家陆港型物流枢纽承载城市重大机遇，制定实施方案，开通和加密至国内重点省会城市的航线航班，争取临时开放延安航空口岸，开通国际航线。加快推进南非、格鲁吉亚延安产业园建设，提高利用外资水平。发挥海关、进出口检验检疫机构作用，推进临空产业园、高新区保税仓库建设。扩大区域合作范围，跟进落实友好城市、企地合作协议事项，推动合作项目尽快落地实施。招商引资到位资金524亿元，引进外资2570万美元，进出口总额达到2.2亿元。

（七）创新社会治理，营造和谐安定有序良好环境。围绕解决影响人民安居、安定、安全的重点难点问题，多措并举，综合施策，打造共建共治共享的社会治理格局。

防范化解矛盾纠纷。强化源头排查化解，建立五级信访网格，推广富县“两说一联”模式，落实领导干部定点接访、重点约访、带案下访、上门回访责任，综合运用法律、政策、经济、行政等手段和教育、调解、疏导等办法，解决重点领域突出信访问题。大力推进网上信访，引导群众合理表达利益诉求，依法规范信访秩序。

筑牢安全生产防线。落实安全生产责任制，夯实属地管理、行业监管和企业主体责任，完善重大安全风险评估机制，强化煤矿、危化品、道路交通、公共消防、森林防火等重点行业和事故多发领域安全生产专项治理，防范遏制重特大事故发生。完善突发事件应急管理机制，加快应急产业发展，增强处置突发事件应急保障能力。

全力维护社会稳定。深化新时代“十个没有”平安创建，完善立体化社会治安防控体系，推进“雪亮工程”建设，加大扫黑除恶专项斗争力度，依法惩治黄赌毒、黑拐骗和侵犯公民个人信息等违法犯罪活动，严惩各类黑恶势力及其“保护伞”，铲土深挖、逐一见底。加大自媒体和网络直播平台监管力度，依法治网管网。依法加强民族宗教事务管理。支持工会、共青团、妇联、工商联、科协、文联、作协等群团组织工作。

深入开展双拥共建，支持部队建设，做好退役军人事务管理，维护军人军属合法权益，巩固和发展军政军民团结局面。

（八）持续保障改善民生，不断满足群众美好生活新期待。坚持以人民为中心的发展思想，时刻关注人民群众普遍关心的痛点难点焦点问题，把顺民意、解民忧、暖民心的实事办好、好事办实。

大力促进就业创业。实施稳就业精准帮扶行动，鼓励创业带动就业，落实创业担保贷款政策，加大创业孵化基地、信用乡村和就业扶贫基地建设。加大人才引进和培养力度，落实人才评价激励保障措施，推进大众创业万众创新。做好高校毕业生、农民工、退役军人、城镇就业困难人员等重点群体多渠道就业工作，城镇新增就业1.5万人，城镇登记失业率控制在4.3%以内。深入推进收入分配制度改革，建立完善工资增长机制，确保城乡居民收入与经济同步增长。

健全社会保障体系。落实全民参保计划，推进被征地农民参加城镇职工养老保险试点，完善城乡居民基本医疗保险制度和大病保险制度。健全农民工工资保障体系。启动“养老服务提升年”计划，推进医养结合、智慧养老，提升居家养老服务水平。统筹推进助残、救孤、济困等福利事业发展，建立乡镇临时救助预备金制度，支持社会力量开展农村留守儿童关爱服务。完善住房市场体系和保障体系，启动“大物业”改革，持续改善群众住房条件。加快市社会福利院、市殡仪馆迁建和3个殡仪服务中心建设。

优先发展教育事业。落实立德树人根本任务，强化学校管理，提升办学质量，加快构建德智体美劳全面培养的教育体系。着力推进教育综合改革，推行校长职级制改革，健全教师激励保障机制，加快新区江苏中学、第三小学和广东中学建设，新改扩建中小学、幼儿园21所，启动聋哑学校迁建。推广宝塔区教育综合改革经验和宜川中学“自主教学”模式。开展校外培训机构专项治理。支持延安职业技术学院创建“国内一流、特色鲜明”优质高职院校，支持延安大学建设更有特色更有水平的综合性大学。

加快健康延安建设。深化医疗联合体及城市公立医院综合改革，加快医养融合，支持社会力量创办高端综合医院和特色专科医院。加强公共卫生服务体系建设，加大基层人才引进和培养力度，完善镇村一体化管理模式，推进分级诊疗。加快健康延安和健康家庭、健康社区、健康学校等健康细胞建设，全面提升城乡居民健康水平。加强食品药品监管，深化国家食品安全示范城市创建。

加快文体事业发展。深入实施文化惠民工程，开工建设延安新闻传媒中心，加快延安博物馆建设和广播转播台迁建，完成基层综合性文化服务中心建设任务。实施“135”艺术创作工程，推进国家级陕北文化生态保护实验区建设。加快新区全民健身运动中心等第十四届全运会场馆建设，完善城乡公共体育设施，深入开展群众性体育活动。

各位代表，“我们要坚持把人民群众的小事当作自己的大事，从人民群众关心的事情做起，从让人民群众满意的事情做起。”今年，认真办好“民生十件实事”：一是拓宽城区易堵道路6处，改造交叉路口9处，新建停车场5处。二是改造城区供水管网7.5公里，设立公共场所直饮水点20处。三是更换城区老旧供热管网3.7公里，扩大集中供热面积180万平方米。四是改造老旧小区6个，新增一批“口袋公园”。五是开工市人民医院改扩建项目，建成投用市精神病医院。六是中心城区新增幼儿园学位1000个。七是创建社区融合服务中心5个，开办社区共享餐厅15个。八是建成跨河便民桥3座。九是新建市区公共卫生厕所20座。十是建成开放陕北说书馆、市图书馆，分时段开放学校体育场馆。

三、全面加强政府自身建设

新时代、新形势、新任务，对政府工作提出了新的更高要求。我们必须增强政治意识，提升履职能力，勇于担当作为，努力建设人民满意政府。

强化政治责任，争做忠诚表率。把政治建设摆在首位，结合“不忘初心、牢记使命”主题教育，扎实开展“讲政治、敢担当、改作风”专题教育，树牢“四个意识”，坚定“四个自信”，坚决做到“两个维护”。敬畏宪法、尊崇法律，深入实施“七五”普法，推进法治政府建设，运用法治思维、法治方式、法治手段行使权力、履行职责。依法接受市人大及其常委会法律监督和工作监督，自觉接受市政协民主监督，主动接受群众监督和舆论监督，提高人大代表建议和政协提案办理质量。

增强履职本领，争做担当表率。加强学习调研，掌握新知识、熟悉新领域、开拓新视野，克服本领不足、本领恐慌、本领落后，增强“八个本领”，锤炼“五个过硬”，提高政府工作的科学性、系统性、预见性。始终保持昂扬向上的进取心，敢担当、勇担当、善担当，风险面前敢闯敢试，矛盾面前敢抓敢管，困难面前敢作敢为，接受任务不讲条件，执行任务不找借口，完成任务不打折扣。

提高服务效能，争做为民表率。加快转变政府职能，凡是市场能自主调节的就让市场调节，凡是企业能干的就让企业干。坚持马上就办、办就办好，持续推进简政放权，着力提高行政效能，创造性推动工作落实。把人民群众的期盼作为政府工作的根本目标，恪守为民之责,善谋富民之策,多办利民之事，始终与人民群众想在一起、站在一起、干在一起，努力奔跑，在奋斗中追梦圆梦。

坚持从严治政，争做廉洁表率。落实全面从严治党主体责任，以永远在路上的恒心和韧劲持续转变作风、纠正“四风”，着力解决重“痕”不重“绩”，留“迹”不留“心”等问题。压减检查考核频次，减轻基层负担。坚持用制度管权管事管人，扎紧织密制度“笼子”，使政府工作人员知敬畏、存戒惧、守底线。强化审计监督，严格财经纪律，严控“三公”经费。坚持零容忍、无禁区、全覆盖，重遏制、强高压、长震慑，驰而不息开展党风廉政建设和反腐败斗争，以实际行动树立为民务实清廉政府形象。

各位代表！实干托起梦想，奋斗铸就辉煌。让我们更加紧密地团结在以习近平同志为核心的党中央周围，在市委的坚强领导下，提振追赶超越精气神，汇聚高质量发展正能量，低调务实不张扬、埋头苦干，以优异成绩庆祝中华人民共和国成立70周年！
